# Study of *Lophomonas blattarum* Infection in Kidney Transplant Patients in Mashhad City, Iran

**DOI:** 10.1155/2020/6631224

**Published:** 2020-12-17

**Authors:** Zahra Gheisari, Fariba Berenji, Fatemeh Nazemian, Seyed Ali Akbar Shamsian, Lida Jarahi, Mahmoud Parian, Bibi Razieh Hosseini Farash, Ghodratollah Salehi Sangani

**Affiliations:** ^1^Department of Parasitology and Mycology, Faculty of Medicine, Mashhad University of Medical Sciences, Mashhad, Iran; ^2^Department of Nephrology, Emam Reza Hospital, Mashhad University of Medical Sciences, Mashhad, Iran; ^3^Department of Community Medicine, Faculty of Medicine, Mashhad University of Medical Sciences, Mashhad, Iran; ^4^Department of Parasitology and Mycology, Emam Reza Hospital, Mashhad University of Medical Sciences, Mashhad, Iran

## Abstract

**Background:**

*Lophomonas blattarum* is a flagellate protozoan which is known as an emerging parasite in the human respiratory system. Organ transplant recipients are considered as immunocompromised patients due to prescription of immunosuppressive drugs. This group of patients is susceptible to opportunistic infection as well as lophomoniasis. This study aims to investigate the prevalence and clinical manifestation of pulmonary infections caused by *L. blattarum* in kidney transplant recipients.

**Methods:**

This is a case-control study including 50 kidney transplant recipients and 50 controls. The sputum samples were collected from 50 kidney transplant recipients with bronchopulmonary infection signs suspected to lophomoniasis admitted in Montaserieh and Imam Reza hospitals, Mashhad, Iran. 50 healthy individuals as the control group were matched for sex and age with case ones. The consent form, checklist, and required information were provided for each patient. All samples were microscopically examined for the flagellated protozoan, *L. blattarum*, using direct smear.

**Results:**

Among 50 kidney transplant recipients suspected to lophomoniasis, *L. blattarum* was identified in sputum samples of 4 (8%) participants of the case group including one female and three males. None of the samples were positive among the control group. Symptoms in patients of this study were high fever (4 out of 4 patients), cough (3 out of 4 patients), and dyspnea (2 out of 4 patients). Three patients showed a positive response to metronidazole treatment.

**Conclusion:**

The results of this study suggest that *L. blattarum* should be considered as a pathogenic agent in kidney transplant recipients. It is necessary to examine sputum samples in posttransplant pneumonia patients, especially in those resistant to antibacterial therapy.

## 1. Introduction

Infectious agents are one of the most common causes of mortality among kidney transplant recipients. Leismaniasis and malaria are the most common protozoan infections in the posttransplantation period [1]. Pulmonary infection in kidney transplant recipients is usually caused by respiratory viruses, CMV, bacterial and fungal pathogens, and *Pneumocystis jirovecii* [2]. Recently, pulmonary infection by *Lophomonas* as an emerging infection has been increasingly considered, especially in immunocompromised patients [3, 4]. This parasite can infect immunocompetent individuals [5] and is more common in patients with immune deficiencies [6]. The first case of lophomoniasis was reported by *Chen* and *Mengsin* in 1993 [7]. The first case of lophomoniasis in Iran was reported in a 31-year-old woman with sinusitis by *Fariba Berenji* in 2015 [8]. After that, other studies were carried out to investigate prevalence and pathogenicity of *L. blattarum* at different ages among the patients with different clinical conditions such as allergic asthma [9, 10], sinusitis [8, 11], and coinfection with tuberculosis [12], immunocompetent and immunocompromised patients [13, 14], and pediatric patients [15, 16]. Sobarzo reported a high prevalence of 35.8% in sputum samples of patients with respiratory disorders [17]. Also, *L. blattarum* was diagnosed in 33.8% of BAL samples by Berenji et al. [5].

Organ transplant recipients are more susceptible to infection because of the use of immunosuppressive drugs that result in mortality and morbidity after transplantation. Therefore, prevention, early diagnosis, and proper treatment of infectious diseases are vital factors in successful transplantation.

*L. blattarum* is usually round, oval pyriform with a length approximately from 20 to 60 *μ*m and a width from 12 to 20 *μ*m. This parasite has a tuft of flagella in which longer ones in the center are surrounded by the smaller ones in the periphery and extend from the anterior pole of the organism. The current diagnostic method of this parasite is based on morphological characteristics in human fresh samples including sputum, bronchoalveolar lavages (BALs), and tracheal aspirates. This multiflagellated parasite may be confused with the ciliated bronchial cells in BAL samples under the light microscope. However, there are some morphological features to differentiate between the parasite and ciliated bronchial cells such as marked terminal bar and columnar shape of epithelial cells, difference in position of the nucleus, and identical length of cilia in epithelial cells versus unequal length of flagella in the parasite [18].

This work was conducted to study the prevalence and clinical manifestation of lophomoniasis in kidney transplant patients and to compare with healthy individuals. The results of this study can provide a new insight to *L. blattarum* as a potential agent for pulmonary infection in organ transplant recipients.

## 2. Materials and Methods

### 2.1. Ethical Consideration

The research proposal has been approved by the Ethics Committee of Mashhad University of Medical Sciences (ethical code no. IR.MUMS.fm.REC.1396.397) in accordance with Helsinki Declaration guidelines. The patients were taken under study after signing the consent forms. Authorizations for children's participation were given by their legal guardians.

### 2.2. Study Population

The sample size was determined based on prevalence and frequency of *L. blattarum* in a study done by Mirzazadeh et al. in Mashhad with 95% confidence level with a margin of error of less than 10% [9].

### 2.3. Data Collection and Analysis

In this case-control study (Nov 2018–Nov 2019), after obtaining the consent form and questionnaire, including demographic data and clinical signs, sputum samples were collected from 50 kidney transplant recipients with pulmonary disorders admitted in Montaserieh and Imam Reza hospitals in Mashhad, Iran (case group) and 50 healthy individuals (control group) who were chosen from healthy members of patients' family. Taking into account the inclusion criteria, patients with a history of kidney transplantation, medicated with immunosuppressive drugs, and who were hospitalized for pulmonary disorders were considered to be in the case group. The use of antiparasitic drugs was considered as an exclusion criterion. The case and control groups were matched for age and sex.

**Figure 1:**
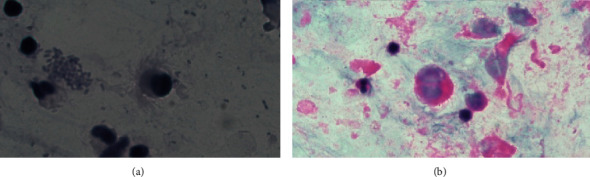
Trophozoite of *L. blattarum* with long flagella in a sputum sample: (a) H&E and (b) Papanicolaou stain.

A nebulizer device was used to facilitate sputum sampling for patients who had difficulty in producing sputum spontaneously. In this method, the patient inhales nebulized hypertonic saline solution, which liquefies airway secretions, promotes coughing, and allows expectoration of respiratory secretions. Sputum induction is simple and noninvasive, and if successful, often precludes the need for bronchoscopy. Sputum samples were examined using direct smear, Papanicolaou, and hematoxylin and eosin (H&E) staining for the parasite (Figure 1). Statistical analysis was performed using SPSS software version 16 (SPSS Inc., Chicago, IL) using Student's *t*-test.

## 3. Results

**Figure 2:**
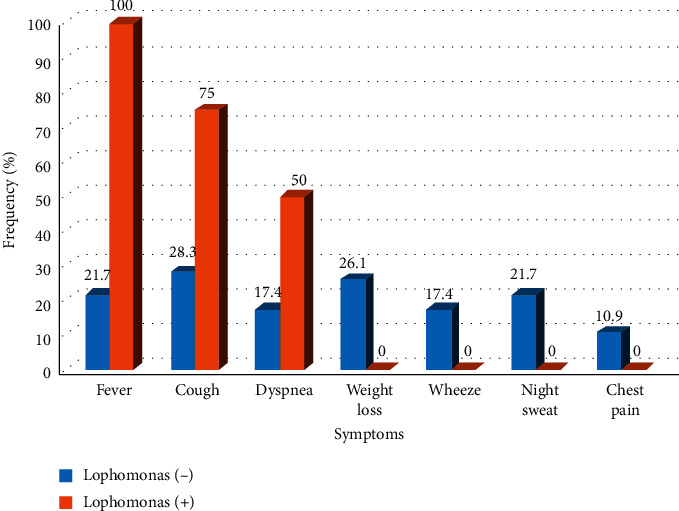
Comparison of clinical signs among patients of the case group with lophomoniasis and without lophomoniasis.

A total of 100 participants were included in this study consisting of 50 kidney transplant recipients and 50 healthy controls. There is not a significant difference of age between patients and healthy control groups. Age average was 48.14 ± 13.47 and 47.74 ± 13.27 for the patient and control groups, respectively (*p*-value = 0.82). In the healthy control group, none of the sputum samples was positive for *Lophomonas*. In the patients group, 4(8%) cases were positive for *Lophomonas* including three males and one female with an age average of 58 years. The length of transplantation was 16.5 years for patients with lophomoniasis versus 14 years for patients without lophomoniasis. Diabetes and cancer were not reported for none of the patients with lophomoniasis. There was no coinfection with HIV in any patients. Also, none of the patients with lophomoniasis declared a history of smoking. The CMV test was positive for one patient. Also, candida was detected in BAL samples obtained from two patients. Among the patients infected with *Lophomonas,* fever, cough, and dyspnea were the main clinical symptoms and were seen in four, three, and two patients, respectively. Weight loss, night sweat, wheezing, and chest pain were not found in any patient infected with *Lophomonas* (Figure 2). Treatment with metronidazole was effective for two patients. One patient died before metronidazole therapy, and no data were available for the latter.

## 4. Discussion

In the current study, *L. blattarum* was detected in 4 (8%) sputum samples of kidney transplant recipients with bronchopulmonary symptoms. Reported prevalence has been varying in different studies. A high prevalence of 40.4% has been reported in children with respiratory disorders during 2016-2017 in Mashhad, Iran [15]. Also, 33.8% of BAL samples were positive for *L. blattarum* in a study published by Berenji et al. [5]. In another study by Mirzazadeh et al., the reported prevalence of the parasite was 10% among 40 asthmatic patients [9], which is almost in accordance with our study. Also, *L. blattarum* was diagnosed in 4 (2.8%) of 142 BAL samples of kidney transplant recipients by Wang et al. [1].

The results of published studies show that clinical signs of lophomoniasis are nonspecific [19]. According to our results, fever (100%) and cough (75%) were reported as common clinical symptoms in patients infected with *L. blattarum* similar to other studies conducted in Iran and China [20, 21]. Zhang et al. through a literature review from 1993 to 2010 reported that cough and fever were present, respectively, in 88.9% and 68.9% of patients with lophomoniasis [20]. Also, cough (87.3%) and fever (28.3%) were reported as the most common respiratory symptoms among children infected with *L. blattarum* in Mashhad, Iran [15]. In another study conducted in Mashhad, Iran, coughing and fever were mentioned as the most common symptoms [21]. Of course, fever was the second most common symptom in these studies, on the contrary of findings in the current study in which fever was the most reported symptom. In another study in the Institution of Nephrology in Jin Ling Hospital, China, high fever (higher than 38 ºC) without cough and breathlessness was recorded in kidney transplant recipients with lophomoniasis during the second to third month after transplantation [1]. In this study, infection occurred during a short time after transplantation, while in our study, a long time has passed over from transplantation of the patients. The results of our study were in agreement with the *Qiang He* study in which fever has been reported in two patients with lophomoniasis who had kidney transplantation more than one year ago [22]. As the highest level of immunosuppression usually occurs during the first six months posttransplantation [23], opportunistic infections are common in this period. Infection with *Lophomonas* a long time after transplantation as well as in immunocompetent individuals [8, 9, 15] implies considering that this organism is not just an opportunistic parasite.

Dyspnea was the third most common clinical symptom (50%) in our study, which is consistent with other studies [21]. Unlike our study, dyspnea was absent among patients included in the *Yang Wang* study [1]. In this study, patients were in the initial period of disease and dyspnea is usually reported in the late period due to disease progression.

Wheezing, one of the clinical symptoms in patients with lophomoniasis, was less reported in previous studies. In the current study, wheezing was not detected in any patient. Nevertheless, in another study conducted in Mashhad, Iran, it was reported as one of the four common clinical symptoms among children infected with *L. blattarum* [15]. Low prevalence of wheezing may be due to the age of the population under study. Wheezing is more common in children because of anatomical factors such as small caliber of airways and also some immunological factors that result in excessive amounts of secretions [24].

Other clinical symptoms such as weakness, weight loss, chest pain, night sweat, chill, and hemoptysis are less common symptoms reported in patients with lophomoniasis and are also not reported in our patients. Also, they are not present in our findings. Absence of these clinical symptoms may be partially helpful to differentiate lophomoniasis from other pulmonary infections. However, sputum samples from patients with lung infection should be examined for *Lophomonas*, especially if the patient's clinical manifestations have not disappeared after treatment for common lung infections.

*L. blattarum* with protease enzyme facilitates other respiratory infections such as TB and fungal and viral infection. Therefore, a mix infection of *L. blattarum* with other pathogens may be observed [5]. Two cases (50%) and one case (25%) of patients of our study had a mix infection with candida and CMV. These results are in accordance to the He et al. study that reported a fungal concomitant infection in a patient with lophomoniasis [22].

Metronidazole is the ideal choice drug and has been proved to be effective for treatment of *L. blattarum* infection as reported in previous studies [21, 25–27]. Similarly, patients in the current study were treated with metronidazole 500 mg b.i.d. for 15 days, and clinical symptoms were gradually ameliorated.

## 5. Conclusion

A prevalence of 8% for *L. blattarum* symptomatic infection among kidney transplant recipients was reported in the current study. Taking into account the relatively high prevalence of cases of lophomoniasis in kidney transplant patients and the growing number of these transplant recipients, it is suggested that they be routinely screened for *L. blattarum* to reduce their morbidity and mortality.

## Data Availability

The data used to support the findings of this study are included within the article.
